# *Listeria monocytogenes* as a Vector for Cancer Immunotherapy

**DOI:** 10.3390/vaccines8030439

**Published:** 2020-08-05

**Authors:** Jorge H. Leitão

**Affiliations:** IBB—Institute for Bioengineering and Biosciences, Department f Bioengineering, Instituto Superior Técnico, Universidade de Lisboa, Av. Rovisco Pais, Torre Sul, Piso 6, 1049-001 Lisboa, Portugal; jorgeleitao@tecnico.ulisboa.pt; Tel.: +351-218417688

**Keywords:** cancer immunotherapy, cancer vaccines, *Listeria monocytogenes* cancer vaccine

## Abstract

Cancer is a wide group of diseases, which was responsible for 9.6 million deaths in 2018. Cancer immunotherapies have become a reality, with the first approval for sipuleucel-T for prostate cancer therapy occurring in 2010. *Listeria monocytogenes* is a Gram-positive bacterium, mostly known as a food-borne pathogen, capable of causing life-threatening and often fatal infections. However, since in the majority of cases the human immune system is able to mount potent innate and adaptive immune responses that control infections by *Listeria monocytogenes*, the microorganism has become an attractive vector for the development of cancer vaccines. The review by Flickinger Jr., Rodeck and Snook (Vaccines 2018, *6*, 48) on the use of *Listeria monocytogenes* as a vector for cancer immunotherapy is described and commented here.

Cancer is a wide group of diseases that can start in virtually any organ or tissue by abnormally growing cells that invade other parts of the body, ultimately leading to death. According to World Health Organization (WHO) data, cancer was the cause of 9.6 million deaths in 2018 [1]. Traditional cancer therapies, including surgery, radiation and chemotherapy, are often non-specific and associated with several stressful and devastating physical and psychological side effects. In the last few years, immunotherapies evolved as promising alternatives to treat cancer. Although the artificial stimulation of the immune system against cancer cells was viewed as a dream by immunologists and oncologists, immunotherapies became a reality with the first approval of sipuleucel-T for prostate cancer therapy in 2010 [2]. This was followed by the approval in 2011 and 2014 of two antibodies for melanoma treatment [3]. 

In the last years, the cancer immunotherapy field has rapidly evolved. Immunotherapies can be classified as passive and active therapies. Passive therapies encompass the administration of monoclonal antibodies, oncolytic viruses and donor-activated lymphocytes activated towards cancer cells [4]. In contrast to passive immunotherapies, active immunotherapies rely on the active stimulation of specific and long-lasting antitumoral immunity [5]. 

The development of bacterial vaccines against cancer is a highly active research field, with expected applications in the near future. The use of bacteria as anticancer agents was described for the first time in 1890 by the New York Memorial Hospital surgeon William B. Coley [6]. Since then, several live attenuated, dead but metabolically active, and genetically engineered microorganisms of the genera *Clostridium*, *Bifidobacterium*, *Salmonella*, *Mycobacterium*, *Bacillus* and *Listeria* have been reported to target cancer cells and to act as anticancer agents [7]. 

*Listeria monocytogenes* is a Gram-positive bacterium, mostly known as a food-borne pathogen, capable of causing life-threatening and often fatal infections, including bacteremia, encephalitis and sepsis [8]. The organism is capable of producing several virulence factors, including the hemolysin listeriolysin O (LLO), the phosphatidylinositol-specific phospholipase C, the ActA protein involved in host actin recruitment and polymerization, and internalins that mediate the adhesion and internalization of the pathogen by host nonphagocytic cells [9]. Nevertheless, in the majority of cases the human immune system is able to mount a potent innate and adaptive immune response that controls infections by *Listeria monocytogenes*, turning the microorganism into an attractive vector for the development of cancer vaccines. In their paper entitled “*Listeria monocytogenes* as a vector for cancer immunotherapy: current understanding and progress”, Flickinger Jr., Rodeck and Snook present a thorough and elegant review on the use of *Listeria monocytogenes* as a vector for immunotherapies against cancer [10]. In the introductory notes, the authors highlight the attractive features of *Listeria monocytogenes* as a vector for cancer immunotherapies, namely the possibility of a repeated administration of the microorganism to boost T-cell responses, the ability of the bacterium to induce both innate and adaptive immune responses, and its ability to act as a potent stimulator of cell-mediated immunity and cytotoxic lymphocytes. After briefly reviewing the main molecular mechanisms used by *Listeria monocytogenes* to cause human infections, Flickinger Jr., Rodeck and Snook review and discuss the development of attenuated strains suitable for vaccination, focusing on strategies involving the deletion of virulence genes, the construction of strains that ectopically express virulence or metabolic genes, and of strains that are killed but metabolically active. Various strategies used for the genetic engineering of strains expressing the virulence factors ActA and LLO, by fusing them with tumor-associated antigens, are also presented [10]. The authors also present quite a useful table summarizing clinical trials using *Listeria monocytogenes* as a vaccination vector. The perspectives presented by Flickinger Jr., Rodeck and Snook for future developments of *Listeria monocytogenes*-based vaccines point out the targeting of angiogenesis proteins and the use of strategies combining classical approaches like radiation and chemotherapy with *Listeria monocytogenes* vaccines [10]. The possibilities of successful *Listeria monocytogenes* cancer vaccines are immense, and there is great hope that such vaccines will contribute to innovative anti-cancer therapies that benefit cancer patients.
